# Supplementary material for: Proximity labeling reveals dynamic changes in the SQSTM1 protein network
Source: J Biol Chem. 2024 Aug 3;300(9):107621. doi: 10.1016/j.jbc.2024.107621 (PMC11401034; doi:10.1016/j.jbc.2024.107621)

Fig2A-Western blots

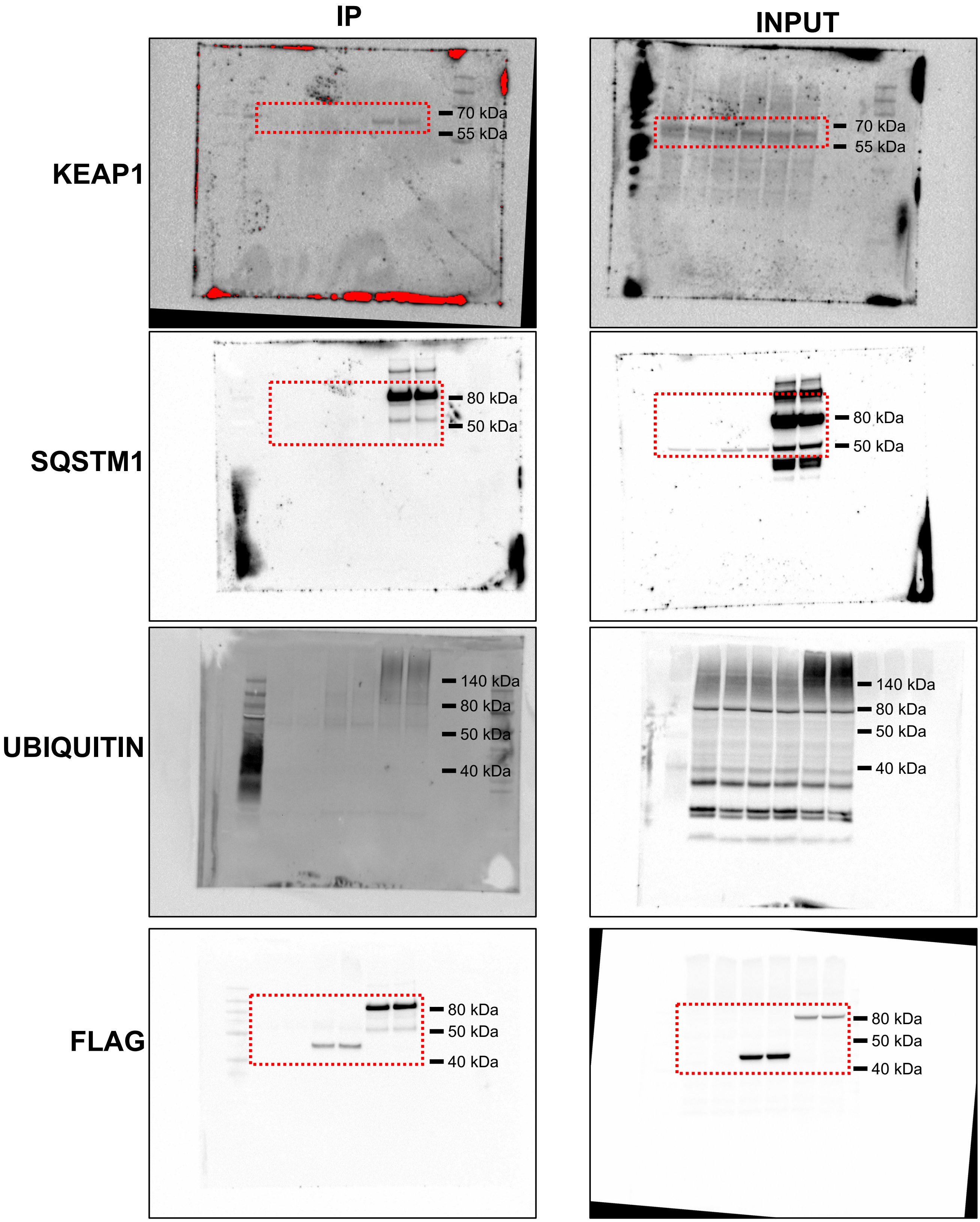

Fig2A-Western blots

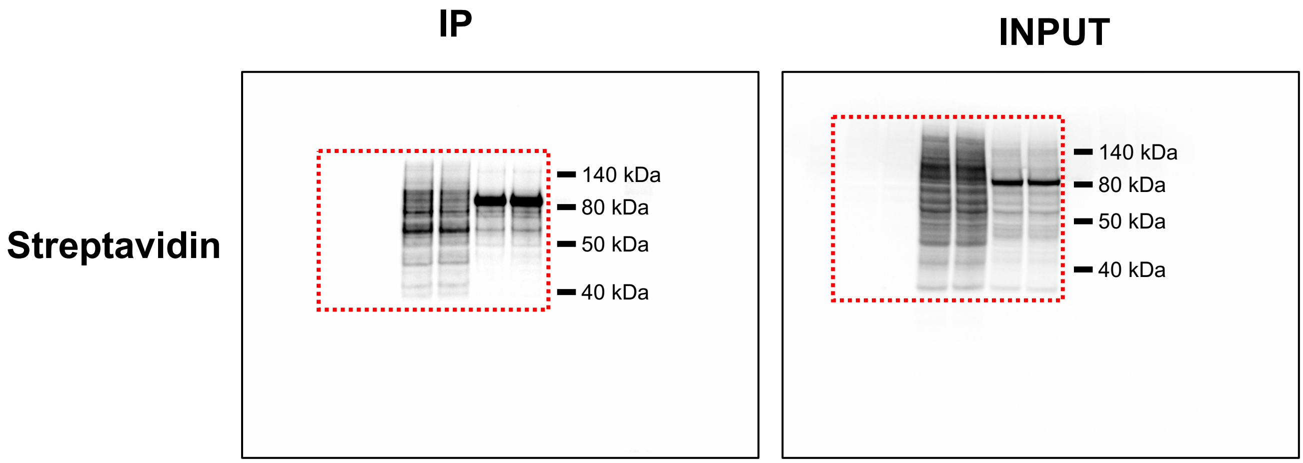

Fig2C-Western blots

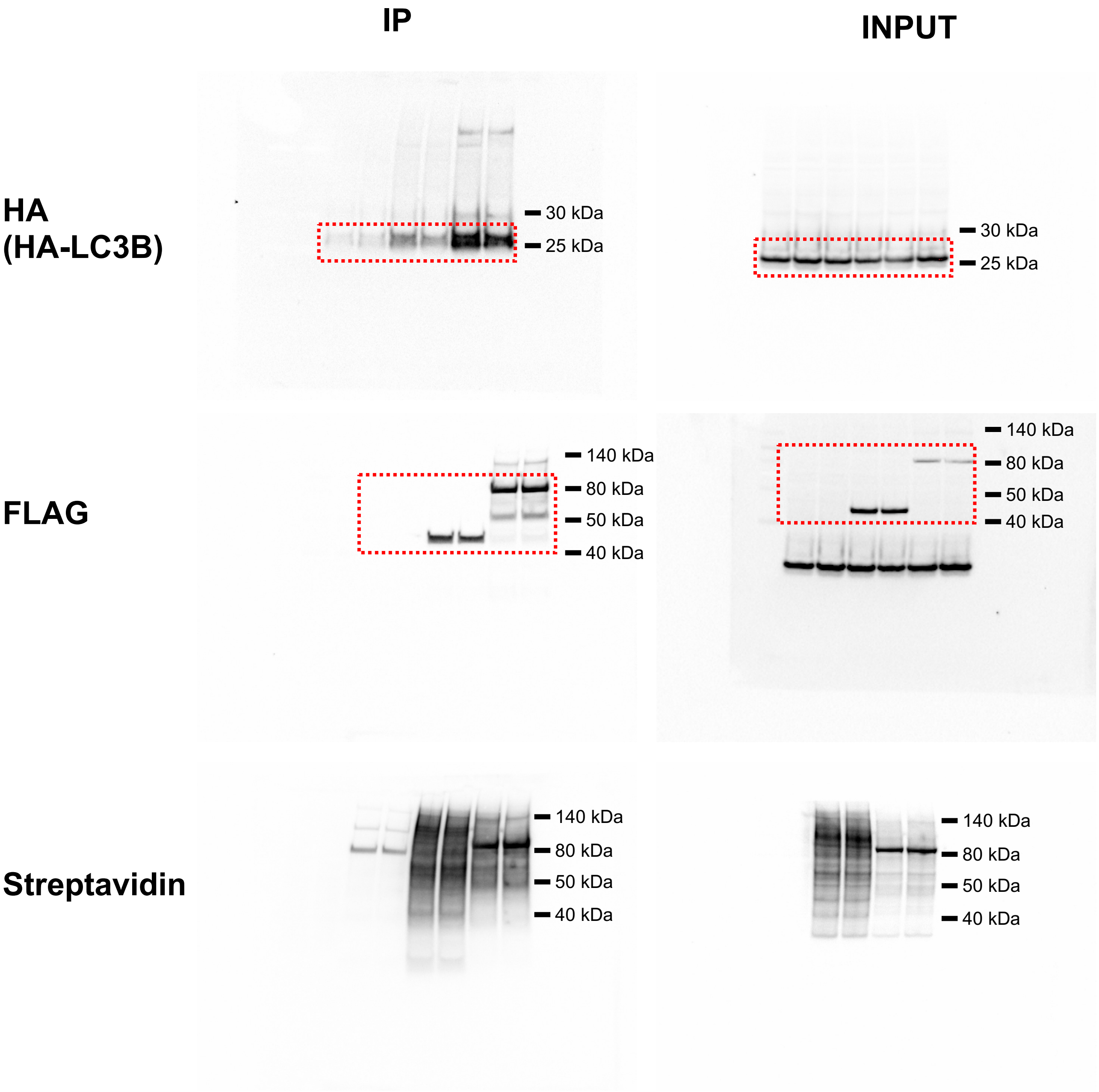

Fig2D-Western blots

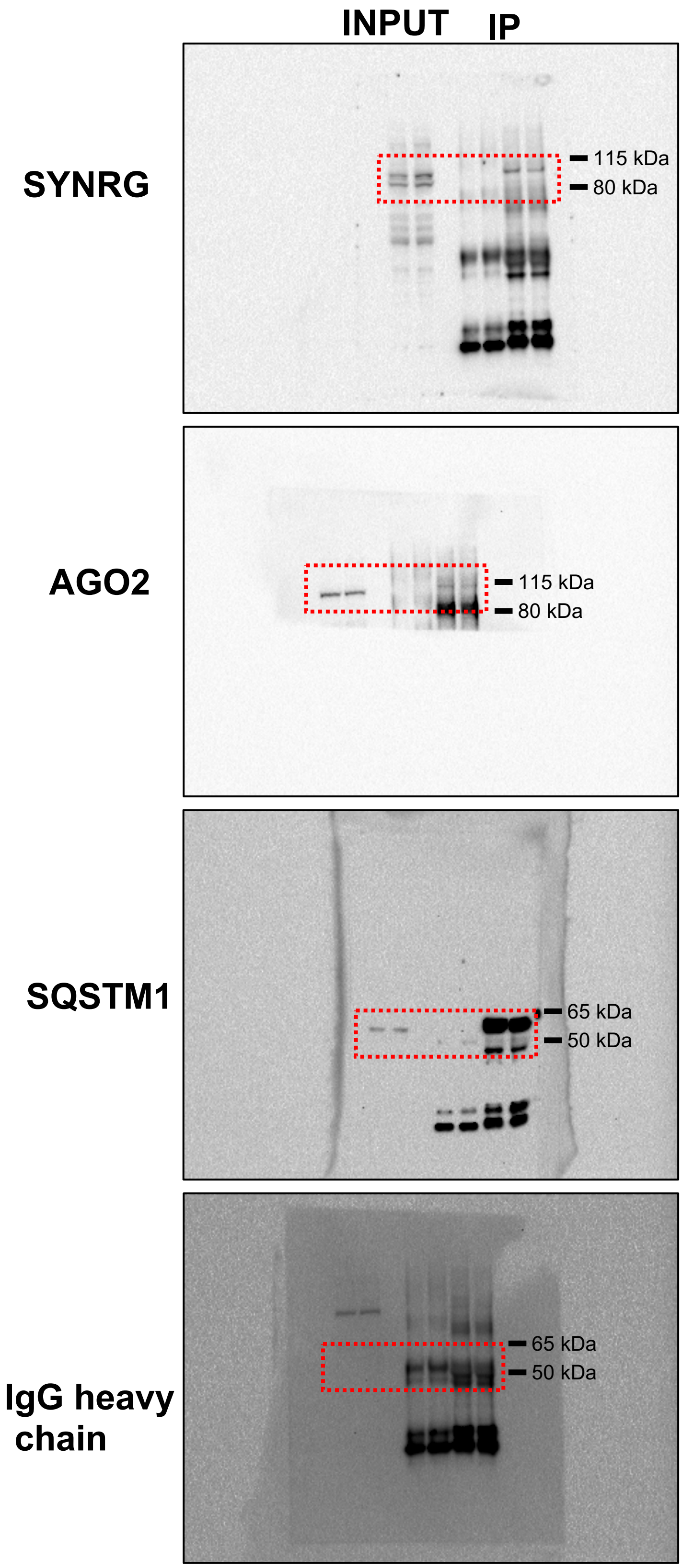

Fig2F-Western blots

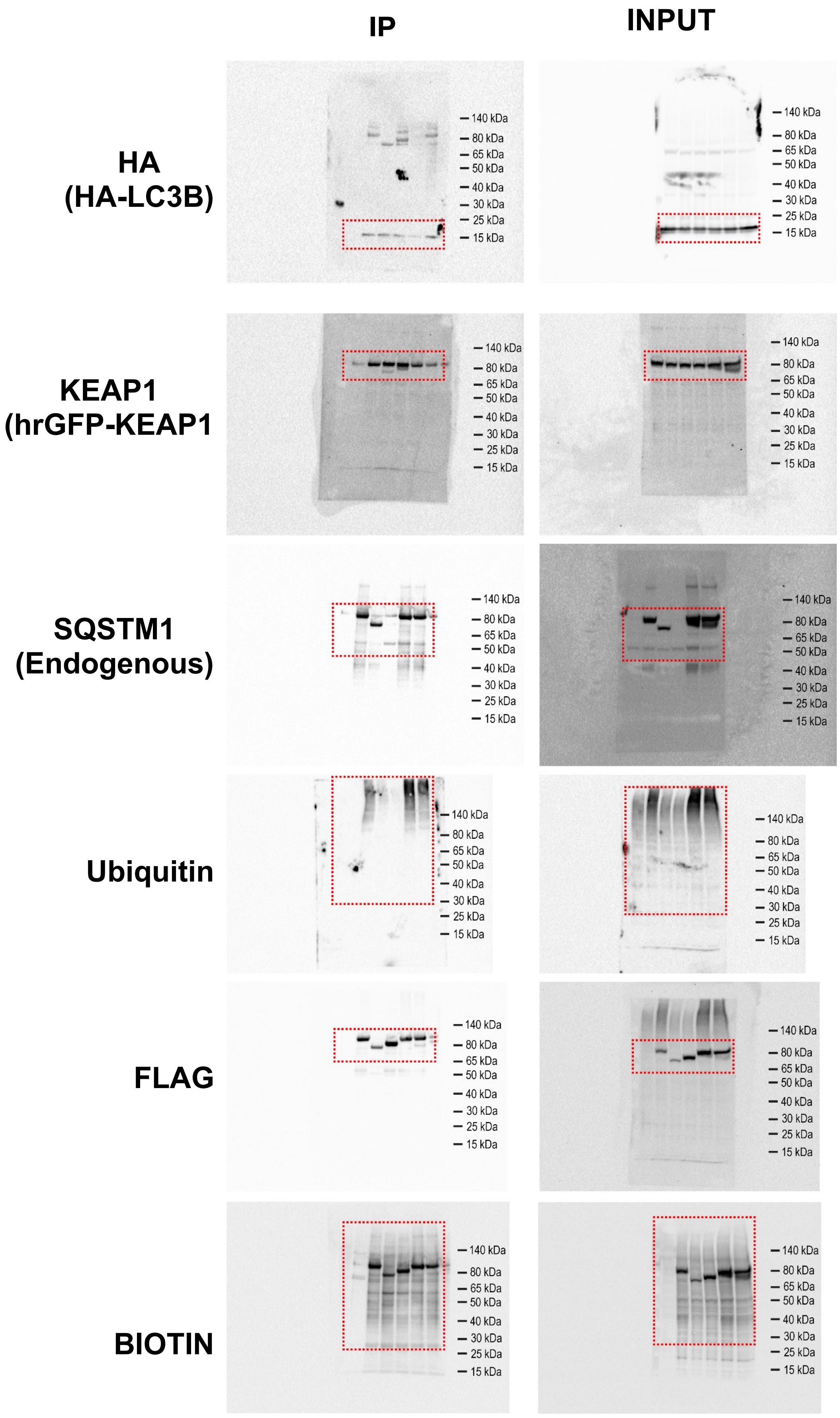

Fig2G-Western blots

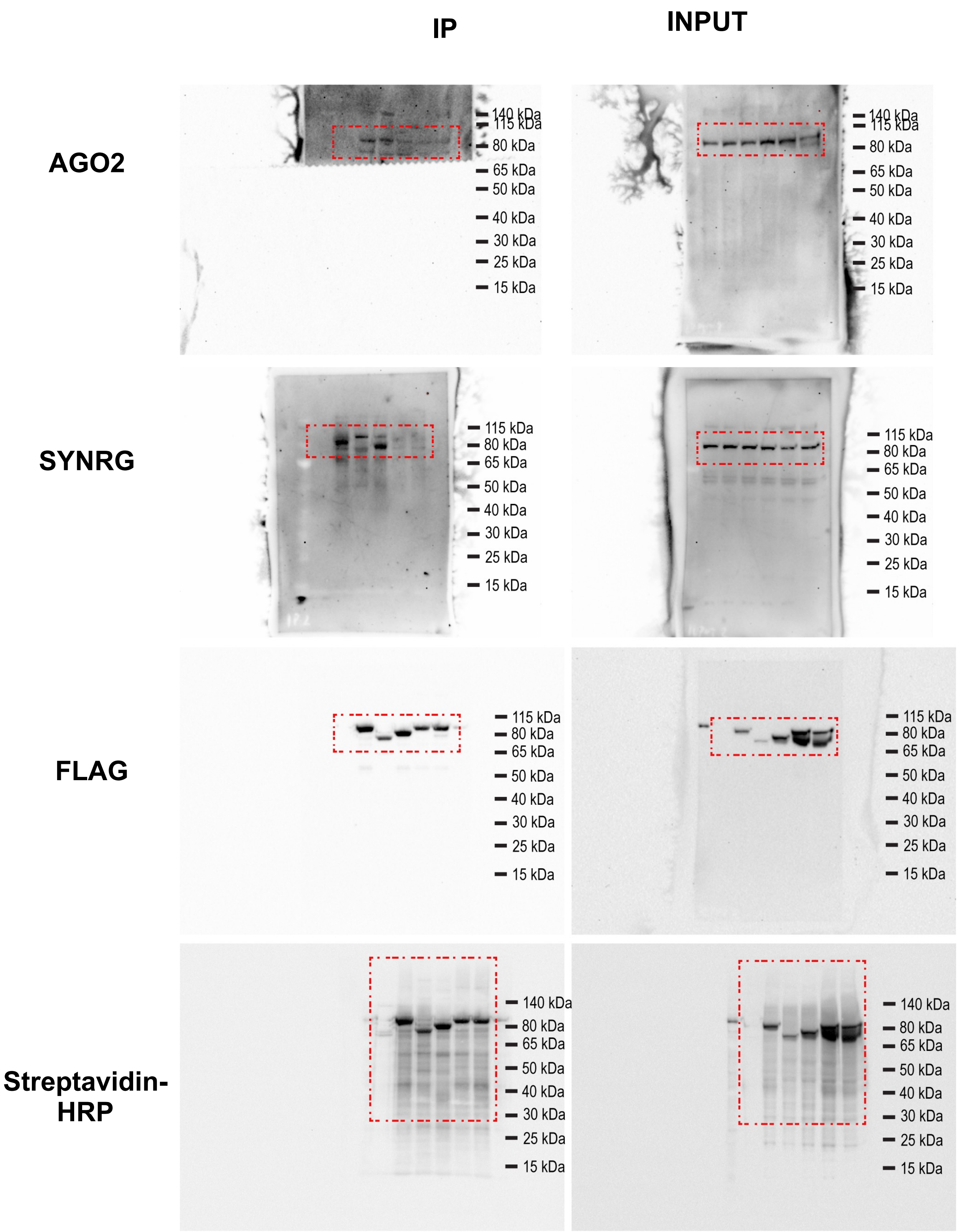

Fig4A-Western blots

FLAG

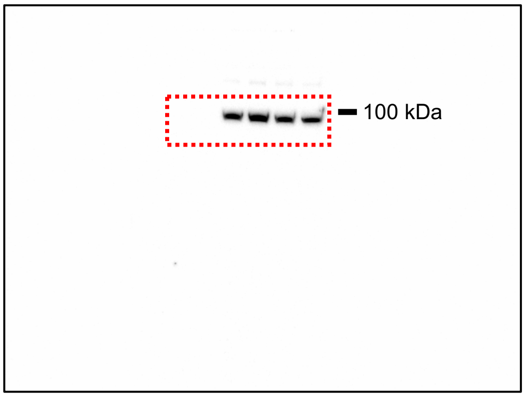

Ubiquitin

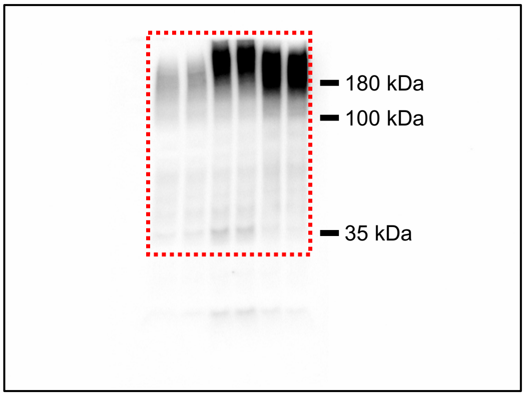

Streptavidin

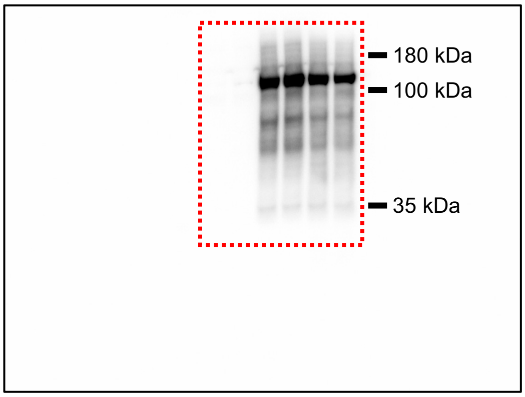

Fig5D-Western blots

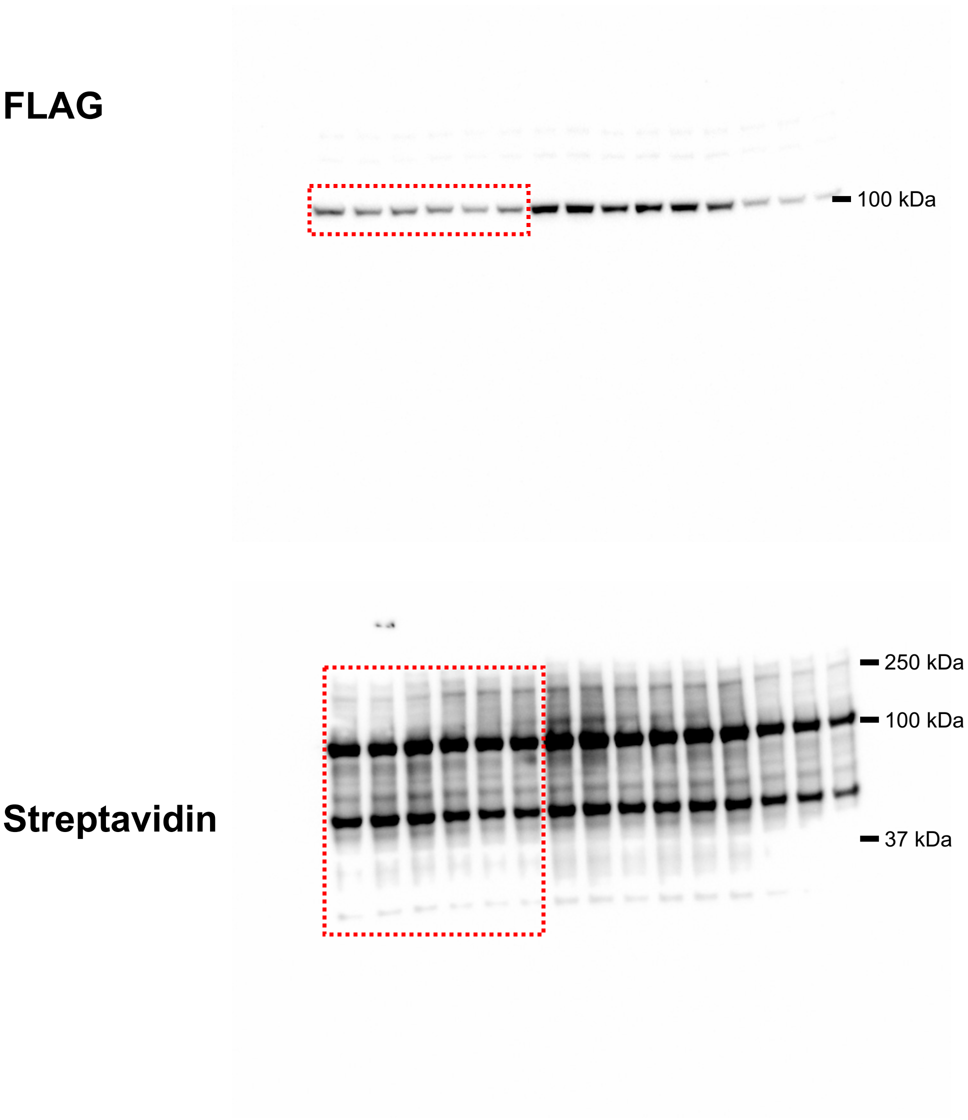

Fig6B-Western blots

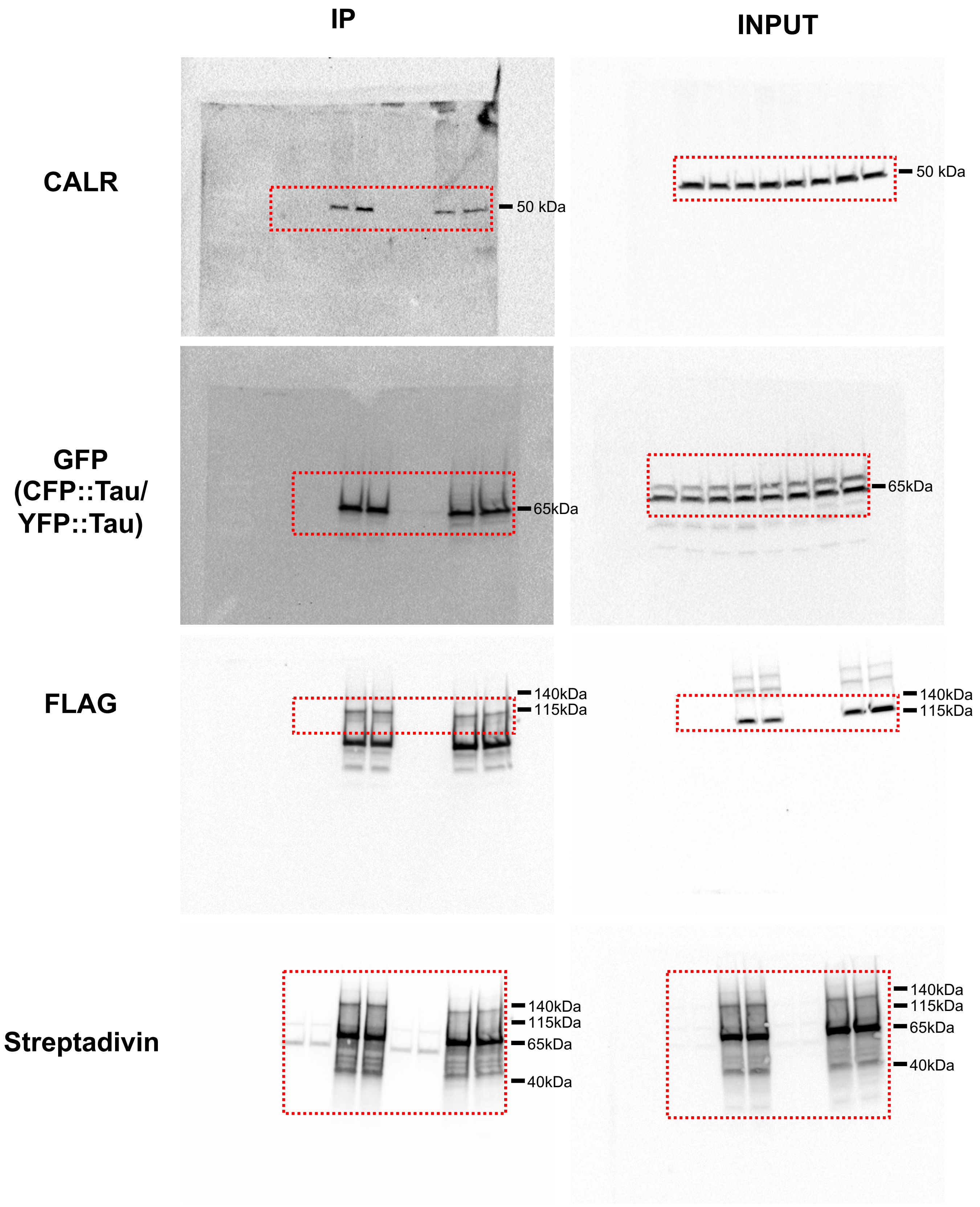

Fig6F-Western blots

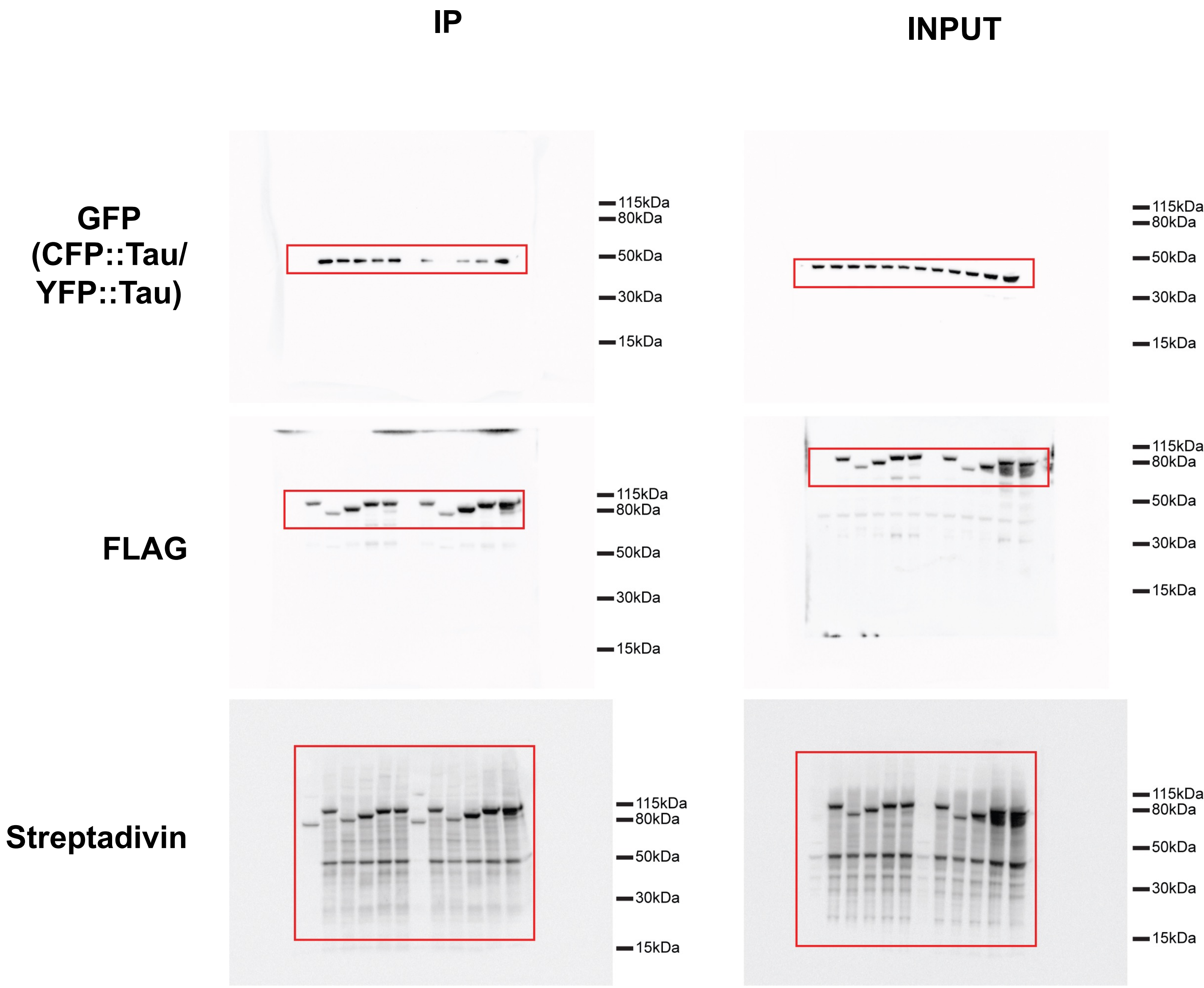

Supplement: Supplemental figures [file mmc1.pdf]
